# The effect of a synthetic cannabinoid agonist (nabilone) on unimodal tactile illusion correlates with a psychometric scores in healthy volunteers

**DOI:** 10.1038/s41598-025-02280-9

**Published:** 2025-05-27

**Authors:** Faiz Mohammed Kassim, Alexander J. W. Davey, Sophie Tod, Jennifer Rodger, Matthew A. Albrecht, Mathew T. Martin-Iverson

**Affiliations:** 1 Center for Social and Affective Neuroscience, Department of Biomedical and Clinical Sciences, Linköping University, Linköping, Sweden; 2 Psychopharmacology Research Unit, School of Biomedical Sciences, University of Western Australia, Perth, WA Australia; 3 Brain Plasticity Group, Perron Institute for Neurological and Translational Science, Nedlands, WA Australia; 4 Experimental and Regenerative Neurosciences, School of Biological Sciences, University of Western Australia, Crawley, WA Australia; 5 Western Australian Centre for Road Safety Research, School of Psychological Science, University of Western Australia, Crawley, WA Australia

**Keywords:** Cannabis, Cannabinoids, Nabilone, Psychosis, Stimulus binding windows, Tactile illusion, Randomized controlled trials, Sensory processing

## Abstract

Our previous studies showed that dexamphetamine, an indirect dopamine agonist, widens Stimulus Binding Windows (BWs) in healthy subjects. The present study aimed to investigate the effect of nabilone, a synthetic cannabinoid agonist, on the BWs in a unimodal illusion: the tactile funneling illusion (TFI). The study also aimed to study the association between tactile illusion with psychometric scores. Healthy participants (n = 32) completed the TFI at various delays and distances of separation of stimuli after receiving nabilone (2–4 mg, PO) or placebo in a randomized, double-blind, counterbalanced, crossover manner. The primary illusory measures were funneling and errors of localisation (EL). Three physiological and five psychometric measurements were also performed. The results showed that nabilone decreased funneling in a delay-dependent manner (*p* = 0.0016), whereby funneling was reduced at 0 ms (*p* = 0.01). Nabilone also significantly reduced EL in a distance-dependent manner (*p* = 0.038). Nabilone increased ratings on two of the five administered psychometric scales (*p* < 0.05), without significantly changing the overall (average) scores. However, there were associations between the overall psychometric scores and funneling under the strongest (0 ms delay) illusion condition, which is dependent on the drug condition (nabilone* ρ* = 0.45, *p* = 0.028). To conclude, unlike the effects of dexamphetamine, low activation of the cannabinoid system decreases the illusory perception of funneling, with narrowing spatial BWs.

## Introduction

There is scientific and health interest in a possible association between cannabis use and psychosis, particularly in schizophrenia, especially with the rise in the use of medicinal cannabis around the world. One approach to investigate the relationship between cannabis and psychosis it to examine the effect of cannabinoids on stimulus binding windows (BWs). Studies showed that multisensory integration is modulated by the physical separation in time or space over which stimuli are presented, referred to as the temporal and spatial BWs^1,2^. Our lab^3–6^ and others^7–12^ have found evidence that people with schizophrenia, or people high in trait schizotypy, have abnormal processing of stimuli and widened BWs (^13,14^ for review). To explore the link between acute cannabis use and psychosis, we performed an experiment studying alterations in tactile perception through tactile illusion tests that manipulate the spatial and temporal separation of tactile stimuli. This might specifically provide insight into tactile hallucinations, which are a commonly experienced subtype of hallucination but remain relatively less well studied.

Illusory procedures have been used previously to investigate body perceptions in people with schizophrenia with passivity symptoms, which are characterized by disturbances in core body representations including body image and body schema (e.g.,^3–5^). For example, the Rubber Hand Illusion (RHI) test indicates increased illusory perception in people with schizophrenia and their offspring^9,15^. Similarly, dexamphetamine administration to healthy volunteers increases illusion strength and widens BWs in the RHI^16^ or Tactile Funneling Illusion (TFI)^17^. Therefore, based on the implication of dopamine in BWs and based on the evidence of the widened BWs in people with schizophrenia, if cannabinoids increase BWs (like dexamphetamine in RH, TFI or schizophrenia), then it can be argued that cannabis may have dopamine dependent pathways. In contrast, if cannabinoids decrease BWs, then acute cannabis may have dopamine-independent pathway to psychosis relative to a chronic psychotic disorder like schizophrenia, as previous studies showed that acute cannabis or tetrahydrocannabinol (THC) intoxication increases psychosis-like symptoms^18–21^. There are no studies showing the effect of cannabinoids on BWs to elucidate the associations between cannabis and psychosis. However, some studies showed that cannabinoids or cannabis use impairs binocular depth perception^22—24^, like the impairment observed in people with psychosis symptoms^25–27^.

Our previous study also showed that dexamphetamine indirectly influenced unimodal illusion through its psychotomimetic effect^28^. Importantly, other studies also showed that there is a positive association between the degree of psychosis-like experiences (PLE) and illusory experience in healthy participants^29,30^. Moreover, there is a concern that baseline personality scores or the degree of PLE (such as schizotypy) may influence the effects of cannabis, and exploring the impact of cannabinoids on psychometric scores and the association between individual differences in psychometric scores (personality traits) and cannabis use is valuable^31^. Studies have also marked that the use of natural cannabis or synthetic cannabinoid use is associated with PLE^32,33^ and other personality traits^34,35^. Therefore, the present randomized trial aimed to investigate the effects of a synthetic cannabinoid agonist (nabilone, 2–4 mg, PO) on the limits of BWs in TFI, while manipulating both spatial and temporal separation of stimuli. The study also aimed to investigate the associations between tactile illusions with psychometric scores before and after the administration of nabilone. We hypothesised that nabilone would change the experiences of unimodal illusory perception or BWs, through its indirect effects on dopaminergic system. We also hypothesized that nabilone would influence a unimodal tactile illusion based on psychometric scores as it has been observed during dexamphetamine administration.

## Methods and materials

### Participant recruitment

The University of Western Australia (UWA) Human Research Ethics Committee granted approval for the experiment (RA/4/20/4558) and the trial has been registered with the Therapeutic Goods Administration Clinical Trial Registry (CT-2018-CTN-02561) and the Australian New Zealand Clinical Trials Registry (ACTRN12618001292268). All methods were performed in accordance with the relevant guidelines and regulations.

Thirty eight healthy participants with no known cannabis hypersensitivity, substance use disorder, neurologic disorders or impairments were recruited through various methods, including lecture announcements, word of mouth, advertisements, and university-group emails. Interested individuals were sent a prospectus package containing the Participant Information Form (PIF), Participant Consent Form, consumer medication information, and contact information. Participants were instructed to abstain from psychoactive substances for 24 h prior to the test. The inclusion and exclusion criteria can be found in our previous report^36^. Lastly, 32 participants, consisting of 12 females, completed the TFI tasks as outlined in the drug and design section. Two of the participants were mixed-handed, and one was left-handed. The mean mean (± SD) age of the group was 25.3 (± 7.0) years. Of the 22 participants who had used cannabis at least once in their lives, 9 had used it more than 10 times.

### Drug and design

The study was a double-blind, counter-balanced, placebo-controlled cross-over study, in which participants received either nabilone or a placebo, with permuted block randomization for drug order. The study consisted of 32 participants in each group, with half receiving the active drug on the first day and placebo on the second, while the remaining half received placebo on the first day and active drug on the second. Nabilone (2–4 mg, PO, MEDA Pharmaceuticals Ltd, UK) was administered for this study. Identical sizes of capsules containing either nabilone or placebo (glucose) were prepared. This dose of nabilone was selected based on previous studies in healthy subjects^37,38^.

A total of 38 volunteers were involved in the study. The first seven participants were administered a cumulative doses of 4 mg (2 mg at 09:30 a.m. and the remaining 2 mg at 11:40 a.m.), but three of them terminated the TFI tasks because of nausea and vomiting. The remaining 35 participants were administered a cumulative dose of 2 mg (1 mg given first and then the remaining 1 mg second) to avoid these side-effect. However, two more participants who received the 2 mg cumulative dose did not complete the tasks due to nausea in one participant and hypotension in the second. One participant withdrew from the trial for unknown reasons. A total of 32 participants completed the TFI tasks.

The testing sessions were conducted between 9:00 a.m. and 5:00 p.m. over 2 days, with a minimum of one-week interval between them. Transport, breakfast, and lunch were provided, and no additional financial incentive was given. Informed consent and medical and psychiatric assessments by psychiatrists were conducted before the experiment on the first day. Demographic information, including sex, age, and weight, was recorded afterwards.

The random allocation sequence was generated by the principal investigator, who allocated participants to each group and had access to information that could identify individual participants during and after data collection. The trial staff involved in participant recruitment and data collection did not have access to the randomization list. The study adheres to CONSORT guidelines.

### General procedures

#### TFI procedure

The illusion test was conducted at approximately 290 min following the first dose. The detailed procedure and corresponding figure for this test can be found in our previous studies^17,28^. The participant was seated and blindfolded, with their dominant arm on the table, palm facing upwards. A straight line was drawn across the arm, five centimeters below the elbow, for reference point. The tests of the tactile using a compass (calliper) began at a distance of five centimeters, with subsequent reductions to one-centimeter increments, ultimately reaching a final spacing of one centimeter (i.e., 5, 4, 3, 2, 1 cm). There were three temporal conditions: 0, 500, and 750 ms. The outcomes measured were funneling, which represents the awareness aspect of illusion (whether the participant perceived one touch instead of two), and error of localization (EL), which measures the error of spatial localization from the reference line (0 cm).

#### Psychological scales

Twenty-five volunteers completed the psychological rating scales on two separate occasions each day, with the initial assessment taking place approximately 90 min and the second assessment taking place approximately 220 min after administration of the first dose of the study drug: The rating scales are Brief Psychiatric Rating Scale (BPRS)^39^, Magical Ideation Scale (MIS)^40^, Perceptual Aberrations Scale (PAS)^41^, Revised Launay-Slade Hallucinations Scale (LSHS-R)^42^ and Scale for the Assessment of Positive Symptoms (SAPS)^43^ were administered. The aforementioned rating scales were administered using either an interview-based or self-report methodology.

#### Physiological measures

Twenty-four participants underwent physiological assessments. Blood pressure (BP), heart rate (HR), and temporal body temperature were recorded at seven points each day, in triplicate, to monitor the effects of the drug and placebo over time. The testing times were conducted at 0, 60, 90, 175, 220, 280, and 370 min post-drug administration.

#### Statistical analysis

The statistical analysis for this study was conducted using R version 3.6.3 (R Core Team 2020) and various packages including dply, ez, mblm, lme4, plyr, and Rmisc. Linear mixed effect model, specifically Type I Analysis of Variance with Satterthwaite’s method, was employed to analyse the effects and interactions of drug, distance, delay, and session, (i.e., day order) on funneling illusion. In order to ensure the residuals approximated a normal distribution, plots of the residuals and Q-Q plots were examined. Paired t-tests were then used for pairwise comparisons between the drug and placebo, while Wilcoxon rank sum tests with continuity correction were employed if the residuals deviated substantially from normality. All pairwise comparisons were conducted with Bonferroni correction. Any exceptions to these methods are detailed in the results.

#### Power analysis

The analysis was conducted using G*Power 3.1 power calculator, and the predicted mean difference (Drug-Placebo) and standard deviation of the difference were based on findings in our lab on the effect of DEX on the “embodiment” component of the RHI, resulting in an effect size of 0.43 with alpha = 0.05 and a power of 0.80 (80%). The minimum sample size required was determined to be 26 (Cohen’s F = 0.34).

## Results

### TFI drug effects

**Fig. 1:**
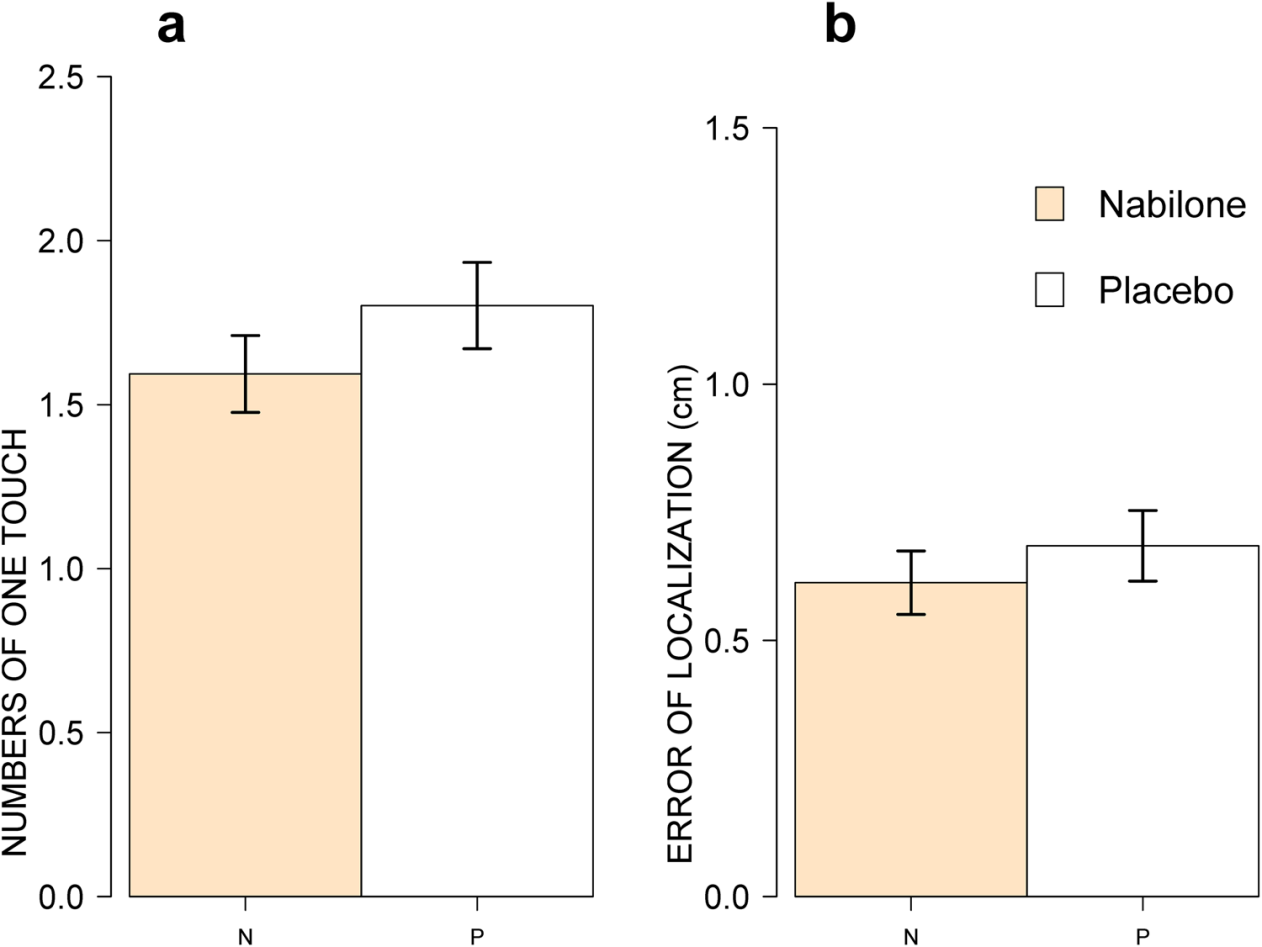
Main effects of nabilone (2–4 mg, PO) on funneling (**a**) and error of localization (**b**) averaged across averaged delay and distance condition. There were no significant main effect of nabilone on funneling or error of localization. N = 32.

Tactile funneling was analyzed with two outcomes: number of times two touches were perceived as one (funneling) and error of spatial localization (EL) away from the reference line. As shown in Fig. 1, there were no significant main effects of nabilone on funneling (F[1, 30] = 1.25, *p* = 0.27) or EL (F[1, 30] = 0.33, *p* = 0.57).

**Fig. 2:**
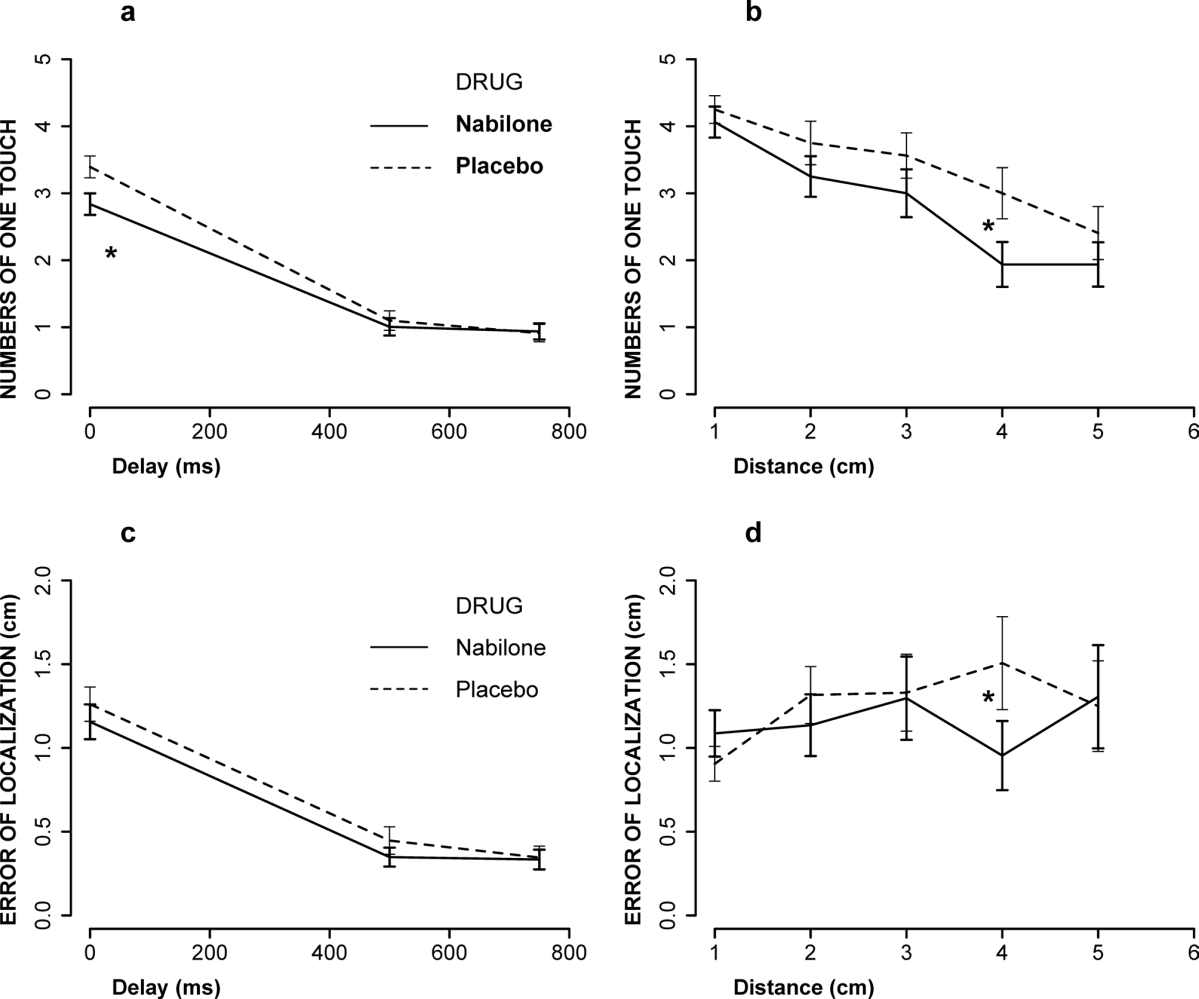
The effect of nabilone (2–4 mg, PO) on funneling (**a**, **b**) and Error of Localisation (**c**, **d**). Data are presented as numbers of one touch (single touches felt) as a function of delay (**a**); numbers of one touch during synchronous delay condition (0 ms) as a function of distance (**b**);  EL as a function of delay (**c**); EL during 0 ms as a function of distance (d). Nabilone significantly reduced funneling illusion at 0 ms (**a**, *p* = 0.013) and at 4 cm during 0 ms (**b**, *p* = 0.02). Nabilone significantly reduced EL at 4 cm during 0 ms (*p* = 0.015). N = 32, for all; ** = *p* < 0.01, * = *p* < 0.05.

As shown in Fig. 2, there is significant interaction between drug and delay (F[2, 660] = 6.4, *p* = **0.0016**); Specifically, nabilone significantly reduced the experience of funneling at 0 ms (*p* = **0.013**). However, the drug, distance and delay interactions was not significant (F[8, 660] = 1.4, *p* = 0.18). For EL, there were no significant interactions between drug and delay (F[2, 660] = 0.6, *p* = 0.54) or drug, distance and delay (F[8, 660] = 1.03, *p* = 0.41). There were no significant interactions between drug and distance for funneling (F[8, 660] = 0.45, *p* = 0.76). However, there were interactions between drug and distance for EL (F[4, 660] = 2.5, *p* = **0.038**).

To assess the impact of the first 4 participants who received the higher cumulative doses (4 mg), we analysed tactile funneling using data from the 28 participants who received the lower cumulative doses (2 mg). Like the outcomes from the overall 32 participants, the linear mixed effect model revealed that there were no significant main effects of nabilone on funneling (F[1, 26] = 1.3, *p* = 0.26) or EL (F[1, 26] = 0.41, *p* = 0.52). There was a significant interaction between drug and delay for funneling (F[2, 552] = 7.93, *p* = **0.0004**), but not for EL (F[2, 252] = 0.66, *p* = 0.51). Unlike the 32 participants, the interaction between drug and distance failed to be significant for EL (F[4, 552] = 2.1, *p* = **0.07**), indicating that there might be either dose or power effect on the BWs.

### TFI non-drug effects

Consistent with a spatio-temporal interaction on illusory perception which showed increasing both delay and distance condition decreases perception of the illusion, there was a significant interaction between delay and distance on EL (F[8, 660] = 4.1, *p* < 0.0001) and on funneling (F[8, 660] = 1.9, *p* = 0.046). There were significant main effects of delay on funneling (F[2, 60] = 113.6, *p* < 0.0001) and EL (F[2, 60] = 48.7, *p* < 0.0001, ƞ^2^ = 0.16). There was also a significant effect of distance on funneling (F[4, 120] = 40.6, *p* < 0.0001), but not on EL (F[4, 120] = 1.3, *p* = 0.29) showing that increasing either delay or distance decreases the experience of funneling, while increasing delay decreases the EL. There were no significant effects of session on funneling (F[1, 30] = 1.1, *p* = 0.3) and EL(F[4 = 1, 30] = 1.8, *p* = 0.29).

#### Psychological effects

We analysed the psychometric measures using data from 24 participants (the overall score was reported previously^36^. As shown in Table 1, Wilcoxon rank-sum test with continuity correction showed significant increasing effects of nabilone on the BPRS (V = 193, *p* = 0.03) and PAS (V = 228, *p* = 0.006), but not on LSHS-R (V = 74, *p* = 0.77), MIS (V = 136, *p* = 0.69) and SAPS (V = 190, *p* = 0.11). There was no significant effect of nabilone on the combined scale score (average-score over all scales) (V = 176, *p* = 0.5).

**Table 1:** Psychological effects of nabilone and placebo (mean ± SD). N = 24.

Psychological effects of drugs						
Scales	Time 1		Time 2		average score	
	Nabilone	Placebo	Nabilone	Placebo	Nabilone	Placebo
BPRS	24.9 ± 2.0	24.5 ± 1.5	25.8 ± 2.8*	24.7 ± 1.8	25.3 ± 2.1*	24.6 ± 1.6
MIS	4.2 ± 4.1	4.5 ± 3.9	4.4 ± 3.7	4.5 ± 4.1	4.4 ± 3.9	4.5 ± 3.9
SAPS	6.2 ± 4.7	5.3 ± 4.5	8.4 ± 9.3	5.4 ± 4.4	7.3 ± 6.4	5.4 ± 4.4
LSHS-R	1.8 ± 1.9	1.8 ± 1.7	1.8 ± 2.0	1.5 ± 1.6	1.8 ± 1.7	1.7 ± 1.6
PAS	3.9 ± 3.6*	3.0 ± 3.1	5.3 ± 7.3	3.2 ± 3.7	4.6 ± 4.8**	3.1 ± 3.4

**Significant at *p* < 0.01; * significant at *p* < 0.05. Time 1: 90 min; Time 2: 220 min.

Significant values are in bold.

#### Psychosis-like experiences (PLE) and TFI experience

Spearman correlation was used to assess how TFI experience associates with PLE scores. There was significant association between PLE scores and funneling during nabilone under the strongest (0 ms) illusion condition (*ρ* = 0.45, *p* = 0.028, n = 24) but not for placebo (*ρ* = − 0.005, *p* = 0.97, n = 24, funneling scores averaged across all spatial and delay conditions). There was no significant association between PLE scores and EL (nabilone and placebo results combined ρ = 0.07, *p* = 0.73, n = 24; nabilone, ρ = 0.30, *p* = 0.14, n = 24; placebo, ρ = − 0.13, *p* = 0.50, n = 24, EL scores averaged across all spatial and delay conditions).

To further assess how changes in experience of the TFI relates to changes in PLE scores, we correlated TFI change scores (drug – placebo) with PLE changes scores. There were no significant relationships between nabilone induced changes in PLE and change in funneling (ρ = − 0.10, *p* = 0.62, n = 24, funneling score averaged across all spatial and delay conditions). There were also no significant relationships between nabilone induced changes in PLE and changes in EL (ρ = − 0.05, *p* = 0.81, n = 24, EL score averaged across all spatial and delay conditions).

#### Physiological measures

**Fig. 3:**
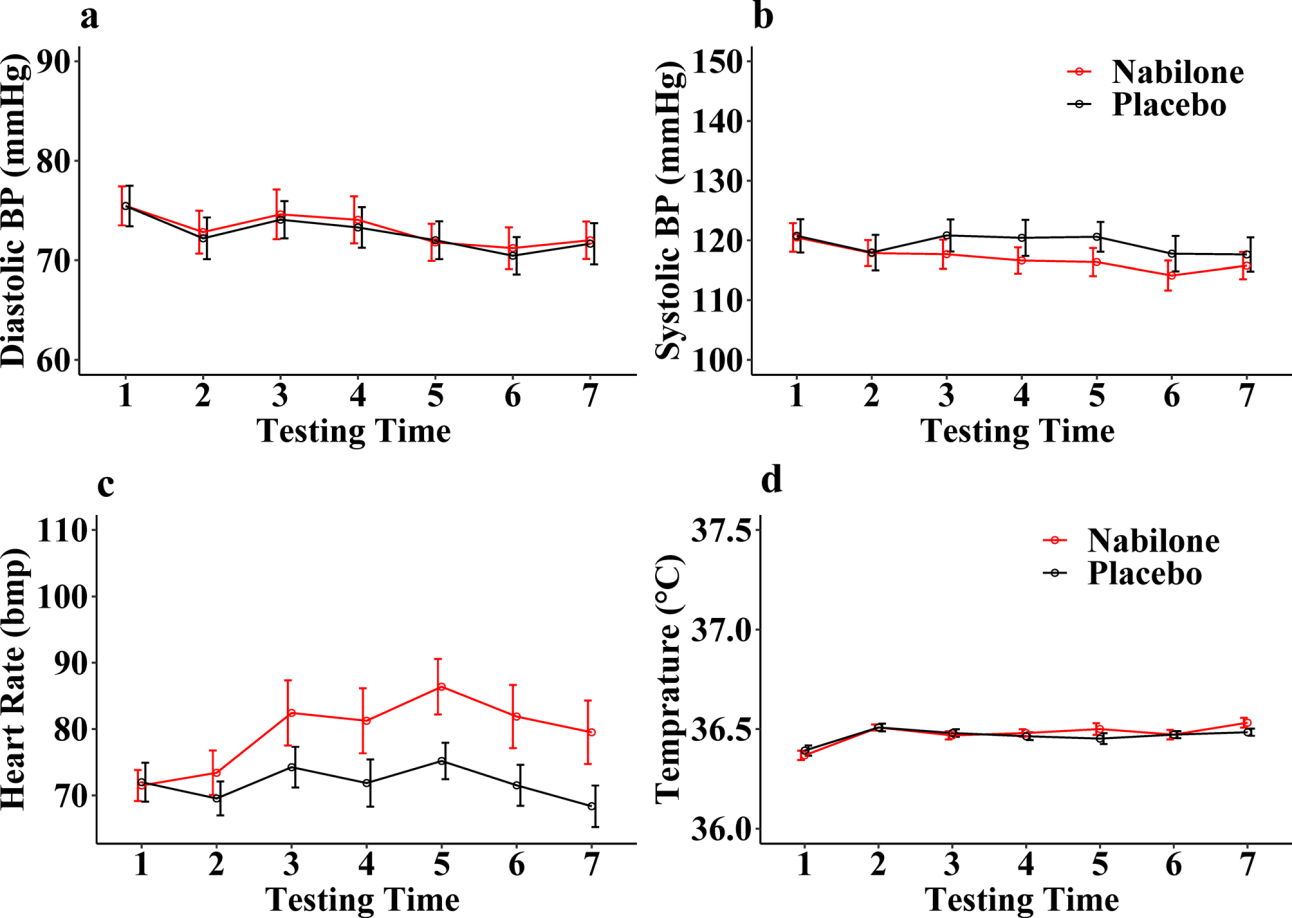
The effect of nabilone (2–4 mg, PO) on diastolic BP (**A**), systolic BP (**B**), heart rate (**C**), and body temperature (**D**) as a function of time. Relative to the placebo, nabilone significantly raised HR (**C**) but not diastolic BP (**A**), systolic BP (**B**), and temperature (**D**). The peak effect of nabilone on HR is at measurement time 5. The seven testing times were 0, 60, 90, 175, 220, 280, and 370 min of post-drug administration. N = 24.

As shown in Fig. 3, analysis of variance (ANOVA) with drug and time as within-subject factors and drug order as a between-subjects factor showed that there were significant main effects of nabilone on HR (F(1, 24) = 10.2, *p* = 0.0039, ƞ^2^ = 0.043) but not on diastolic BP (F(1, 24) = 0.22, *p* = 0.64, ƞ^2^ = 0.0004), systolic BP (F(1, 24) = 3.1, *p* = 0.09, ƞ^2^ = 0.009) and temperature (F(1, 24) = 0.78, *p* = 0.38, ƞ^2^ = 0.002). There were also significant interactions between drug and time on HR (F(6, 144) = 4.8, *p* = 0.0002, ƞ^2^ = 0.012) but not on diastolic BP (F(6, 144) = 0.12, *p* = 0.99, ƞ^2^ = 0.0003), systolic BP (F(6, 144) = 1.5, *p* = 0.17, ƞ^2^ = 0.003) and temperature (F(6, 144) = 1.1, *p* = 0.35, ƞ^2^ = 0.014).

## Discussion

We investigated the role of a synthetic cannabinoid on tactile BWs by administering nabilone (2–4 mg) to healthy individuals undergoing the TFI. For the primary and secondary analysis, we found: 1) Nabilone significantly decreased the funneling illusion during the synchronous condition (0 ms delay) and decreased localization at 4 cm indicating that nabilone narrows spatial BWs; Overall, the results indicate that, unlike dexamphetamine, nabilone does not widen SBWs or TBWs. 2) nabilone increased scores on the BPRS and PAS, suggesting an increase in psychosis-like experiences (PLE), but did not significantly affect SAPS, MIS, or LSHS-R; 3) there was a positive relationship between PLE and experience of the funneling illusion that was present during the nabilone condition, but not the placebo condition; and 4) nabilone increased heart rate but not the other physiological measurements.

Previous experimental studies (e.g.,^18–21^) have shown acute cannabis use can produce a brief intensification of some psychotic symptoms and aggravation of cognitive deficits in otherwise healthy controls and in people with schizophrenia (see^44^ for review). The common psychosis measures like the SAPS^20,45^ or Psychotomimetic States Inventory^18,46^, show an increase in transient psychotic-like symptoms following acute intoxication. Importantly, the study conducted by D’Souza and colleagues^20^ on healthy volunteers reported that THC transiently induces psychotic symptoms. However, psychosis is a complex subjective experience and we might better understand the phenomenology of psychosis by including a broader suite of scales to measure PLEs (e.g.,^47^). In our study, nabilone induced PLEs on two (BPRS and PAS) of the five scales, and there was no significant effect on the average of five scales. This suggests that there is some sensitivity of the cannabinoid experience to a general negatively valenced subjective experience, but there may be less sensitivity to the more specific construct of positive symptoms. It may also be argued that any significant change in subjective state may result in increases in psychosis-like measures, as in for example religious experience^48^, nightmares^49^, and barbiturates administration^50^. Despite having a range of measures to identify the psychosis state, there are limitation to the psychometric measures. First, the questionnaires are liable to self-report bias^51^. Second, it appears that those who report PLE when taking drugs might have high schizotypy to begin with^52^. Further research should clarify whether there is a clear dissociation between subjective experiences reflected by these scales following cannabinoid administration.

### Nabilone decreased funneling illusion

The present study showed that nabilone significantly decreased the funneling illusion, during synchronous stimulation (0 ms delay) and EL at 4 cm, indicating that nabilone narrows BWs for single touches felt, at most, or, at least, nabilone increases tactile sensitivity. However, nabilone did not change temporal BWs, as shown by practically no difference between nabilone and placebo for any temporal delay. However, this may be limited by floor effects. The present findings based on tactile measurements suggest different causal mechanism between psychosis and low level CB1 activation compared to that seen in schizophrenia or dopamine agonism as indicated by narrowing of spatial BWs and a decrease of funneling at synchronous stimuli presentations. In our previous two studies using an indirect dopamine agonist in healthy subjects, we reported an increase in the experience of the illusion and a widening of BWs on multimodal^16^ and unimodal illusions^17^. In addition, studies in schizophrenia (e.g.,^15,29^) have reported widening of temporal BWs on multimodal illusions which might be linked to increased dopamine based on the dopamine hypothesis of schizophrenia.

How exactly nabilone or other cannabinoids modulate the dopaminergic system is not clear. Several studies have shown that THC and synthetic cannabinoids that stimulate cannabinoid receptors (CB) 1 also enhance dopaminergic transmission in the meso-prefrontal loop (^53,54^, see^55^ for review^56–60^). However, non-selective agonists also activate CB2, which might have different indirect effects on the dopaminergic system^61^. It is also possible that dopamine-independent mechanisms (for review, see^62^) play key roles in the effects of nabilone on the reward system, cognition, and perception. The Glu, GABA or cholinergic effects of CB agonists such as nabilone might have important roles. In support, the evidence for regulation of these neurotransmitters (NTs) by CB1^63–71^ is much stronger than is the evidence that the NTs modulate dopamine release (for review, see^72,73^).

Recent findings generally suggest that activation of CB2 might have antipsychotic properties [for review, see^74^. Studies^61,75^ showed functional effects of CB2 on neural networks that include dopaminergic neurons in mice, suggesting that CB2 may have active roles in modulating psychomotor behaviours^76^ and behavioural effects of psychostimulants^61^. Animal studies revealed that CB2 agonists inhibit ventral tegmental area (VTA) DA excitability and modulate dopaminergic behaviours^77,78^. Based on GWAS analysis results, CB2 genes were also associated with PD^76^, indicating that higher CB2 functions may abnormally suppress dopaminergic activity in humans. On the one hand, it has been suggested that lower CB2 function is associated with increased risk of schizophrenia, or it may be harmful, at least in the presence of other risk factors for schizophrenia (see^79^ for review^80^). On the other hand, various studies^81–83^ showed that inhibition of CB1 is associated with reduction of the dopaminergic system. Therefore, the decrease in the funneling illusion after nabilone administration might be due to CB2, while the increased PLE experience might be through CB1 activation.

Previous novel findings^84,85^ that used a cannabinoid agonist and antagonist in clinical schizophrenia studies do not support the cannabinoid hypothesis of schizophrenia. Meltzer and colleagues reported that a cannabinoid antagonist (rimonabant) failed to reduce the psychotic symptoms in people with schizophrenia or schizoaffective disorder^84^. Furthermore, a synthetic cannabinoid agonist (dronabinol) improved core symptoms of the psychosis in a small group of patients^85^, despite the important study limitations. These findings show that, in contrast to the cannabinoid hypothesis of schizophrenia, cannabinoid agonists might have the potential to improve symptoms of psychosis, while cannabinoid antagonists do not, such as in subgroup of patients or in subgroups of patients with treatment-refractory schizophrenia although concerns (such as drug abuse, dose-response relationships, and cognitive adverse effects) following cannabinoid use are major caveats to be addressed^36^.

There are no previous studies investigating the effects of cannabinoids on BWs and the funneling illusion. However, there are studies on the perceptual effects of cannabis or THC based on objective assessments^18,22–24^. These studies reported negative (i.e., more resistant to the illusion) effects of cannabinoids on a visual illusion (binocular depth inversion)^22–24,86^ and the auditory speech illusion^18^. Distortions of these same visual and speech perceptions are also found in people with schizophrenia^25–27,87–91^ and in ultra-high risk for psychosis participants^90,92,93^. In contrast, in the present study, we found that nabilone significantly reduced the perception of the funneling illusion. These discrepancies might be because of the difference in the types of the illusions: the previous negative impacts of cannabis were on visual and speech illusions, indicating that the present effects we describe might be specific to tactile illusions.

### PLE and illusory perception

The present results showed that there was a relationship between unimodal illusory perception and PLE scores, although the baseline PLE scores did not influence the effect of nabilone on TFI. Previous reports in healthy individuals have shown a relationship between PLE scores and illusion experiences. For example, there was a positive correlation between PLE scores and the experience of the RHI in healthy participants^29,30^. There was also a strong correlation between PLE and speech illusion in healthy adults^90^ and in children from the general population^93^. Gonzalez de Artaza and colleagues also found a correlation between PLE and speech illusions, although the correlation was not replicated after adjusting for cognitive ability, sex and age^94^. Some other studies found no apparent correlation between speech illusions and PLE in the general population^89,95,96^. Graham and colleagues reported that schizotypy in healthy subjects correlated with intentional binding (“the subjective contraction of time between a wilful action and the sensory consequences of that action”)^97^. In addition, there was a positive correlation between the severity of psychotic symptoms and RHI experiences in people with schizophrenia^15,98^. Catalan and colleagues reported a positive correlation between PLE and speech illusion in people with schizophrenia^89^. Our result showed that nabilone-induced PLE is associated with the strength of funneling illusion experienced, also indicating that more PLE is manifested in subjects who experience strong funneling illusion.

### Strength and limitations of the study

The important strengths of the present study was its study design and its use of the temporal and spatial conditions to examine BWs, using a cannabinoid agonist. The study also used volunteers whose ages and education levels were very close, which make the participants a homogeneous population and avoid or decrease age-related cognitive differences^99,100–103^. A limitation for this study was the sample size, which affects the precision of the findings, although this limitation was partially remediated by a within-subject design. Increasing the sample size may allow a better separation of psychosis measures and perhaps better identify interaction effects, especially for the three way interactions. Second, the self-reports about recent substance use were not verified by urine tests. However a previous study in our lab that recruited participants from the general community showed that self-reports regarding recent cannabis use are consistent^104^. Third, we used a low-moderate dose of nabilone. Higher doses will have a more robust subjective effect, but it is unclear how this may impact the primary spatial and temporal BW measures. We suggest that both lower and higher doses will need to be considered to elucidate the association between perception and nabilone. Fourth, we used non-selective cannabinoid agonist. Future studies that use selective CB1 and CB2 agonists may identify the mechanisms of cannabinoid-induced PLE and illusory experiences. Lastly, most participants (22) had a history of cannabis; 9 of them consumed cannabis > 10× in total. As cannabis use history might affect the outcomes, future studies might consider comparing cannabis naive with frequent cannabis users.

## Conclusions

Unlike the effects of dexamphetamine in our previous studies, low activation of the cannabinoid system decreases the illusory perception of funneling, with a narrowing of spatial BWs. However, nabilone increased ratings on measures of psychosis-like experiences suggesting that nabilone may render opposite effects on PLE scores and objective illusory assessments that are to be considered and compared in future studies. In addition, there were positive associations between PLE scores and experience of the funneling illusion that was present for nabilone but not placebo.

## Data Availability

Data are available from the corresponding author up on reasonable request.

## Declarations

### Clinical trial registry name

Nabilone and caffeine effects on the perceptions of visually, auditory, tactile and multimodal illusions in healthy volunteers.

### Clinical trial registration number

CT-2018-CTN-02561-1 (Therapeutic Goods Administration Clinical Trial Registry; Date: 18/07/2018) and ACTRN12618001292268 (The Australian New Zealand Clinical Trials Registry; Date: 31/07/2018).
